# Endovascular management of acquired uterine vascular anomalies

**DOI:** 10.1186/s42155-025-00582-7

**Published:** 2025-10-14

**Authors:** Sivert Kupfer, Christian Haslinger, Thomas Pfammatter

**Affiliations:** 1 Institute of Diagnostic and Interventional Radiology, University Hospital Zurich, Zurich, Switzerland; 2 Department of Obstetrics, University Hospital Zurich, Zurich, Switzerland

**Keywords:** Uterine arteriovenous anomaly, Uterine arteriovenous malformation, Uterine artery embolization, Postpartal uterine hemorrhage

## Abstract

**Objectives:**

To evaluate clinical presentation, imaging features, embolization techniques and their outcome for acquired uterine vascular anomalies (UVA) related to obstetric events.

**Materials and methods:**

Thirteen women (mean age = 34; range = 20–40 years) who had undergone interventional radiological treatment of UVAs between 2013 and 2024 were retrospectively analyzed. All patients had a history of an obstetric event. They presented with ongoing postpartal vaginal blood losses (*n* = 11) or were asymptomatic (*n* = 2). Fertilization had been performed by intracytoplasmic sperm injection (ICSI) in 3/13 women. 7/13 women had delivered healthy babies. 6 women had surgical, drug-induced or missed abortions. Postpartum dilatation and curettage had been performed in 4 women. The delay between the obstetric/gynecological event and the radiological intervention ranged from 19 to 193 days (median = 49 days). Long-term follow-up was available in 12/13 patients (median FU = 2.4 years).

Unilateral selective transcatheter embolization was performed in 7/12 patients (n-Butyl-Cyanoacrylate-Lipiodol mixture [BCAL], *n* = 5; trisacryl gelatine particles, *n* = 2); Bilateral uterine artery embolization was performed in 5/12 women (unilateral BCAL combined with contralateral particles in 3/12, or bilateral gelatine sponge slurry in 2/12). In one patient percutaneous direct injection of BCAL into a uterine artery branch pseudoaneurysm was performed.

**Results:**

Primary clinical success without complications was achieved in 10/13 interventions. Re-embolization was successful in the 3 patients with ongoing bleeding despite uterine artery embolization. Follow-up information was available in 12/13 patients (median FU = 2.4 yrs). The pregnancy rate after embolization was 8/12women with a birth rate of 6/8 pregnancies.

**Conclusion:**

Embolization of acquired UVAs is an effective and safe treatment. Preservation of uterine function for future pregnancy after uterine transarterial embolization seems warranted.

## Introduction

Uterine vascular anomalies (UVA) are hypervascular lesions with direct connections between uterine artery branches and the myometrial venous plexus [1, 2]. They can be acquired or congenital. Congenital UVAs, also called arteriovenous malformations (AVMs), have a central nidus with multiple feeding arteries and draining veins. They may extend beyond the uterus. In contrast to AVMs, acquired UVAs have no nidus and an intramural direct fistulous connection between arteries and veins [3]. They usually occur after a recent delivery, abortion or other uterine trauma such as myomectomy, dilation and curettage (D&C), or cesarean section. Rarely, their etiology is a pelvic trauma, a neoplasm, an inflammatory lesion or trophoblastic disease after treatment with chemotherapeutic agents [4, 5]. Regarding their clinical presentation, their imaging features and their interventional treatment there is overlap between the terms “retained products of conception”, “acquired uterine vascular malformation” and “subinvolution of the placentar bed”, justifying the umbrella term “acquired uterine vascular anomaly” [6, 7]. For acquired vascular anomalies the incidence in pelvic sonographic examinations is between 0,6% and 4,5% [2, 8].

Transvaginal ultrasound (TVUS) with color and spectral Doppler is usually the first diagnostic imaging technique for UVAs because it is inexpensive, readily available and safe. Differentiation of UVAs from other vascular lesions, such as retained products of conceptions (RPOCs), can be difficult at times. Therefore, other imaging modalities, such as computed tomography angiography (CTA) or magnetic resonance (MR) angiography, may be useful to further characterize the UVA and plan their treatment. Digital subtraction angiography (DSA) is rarely performed as stand-alone diagnostic tool and is reserved for women undergoing transarterial embolization (TAE) [3, 6, 9].

UVAs can lead to severe abnormal uterine bleeding and even anemic shock. The therapeutic options for UVAs range from conservative therapies such as treatment with hormones to TAE and more invasive treatment options like transvaginal removal of the UVA to uterine artery ligation or hysterectomy [3]. The decision on treatment is based on clinical presentation supplemented by laboratory and imaging results and patient preference, especially if fertility preservation is desired for future pregnancy [6].

The aim of the study was to evaluate arterial embolization of UVAs related to a prior obstetric event with regard to efficiency, complication rate, post-interventional pregnancy and birth rates.

## Materials and methods

This retrospective single-center study was approved by the local ethics committee. A general informed consent for the use of anonymized data for research purposes was obtained from all patients.

Thirteen consecutive women (mean age = 34 years; range = 20 to 40) who had undergone uterine arterial embolization of acquired UVAs at a tertiary referral hospital between 2013 and 2024 were included (Table 1).

**Table 1:** Patient characteristics

Patients, n (%)	13 (100)
Age (years), mean ± SD	33.8 ± 5
Obstetric/gynecological event	
Intracytoplasmic Sperm Injection	3 (23)
Vag. delivery followed by D&C, n (%)	4 (30)
Normal vaginal delivery, n (%)	2 (15)
Spontaneous abortion, n (%)	3 (23)
Medical abortion, n (%)	2 (15)
Surgical abortion, n (%)	1 (8)
Cesarean section, n (%)	1 (8)
Clinical presentation	
Vaginal bleeding, n (%)	11 (85)
UVA without bleeding, n (%)	2 (15)
Time between obstetric/gynecological event and radiological intervention	
Time range(days)	19 to 193
Median (days)	49

Of the 13 patients included, 11 presented with vaginal bleeding and the remaining two patients were diagnosed of having a UVA on ultrasound and MRI at postpartal follow-up examinations. These two patients desired future pregnancies. In three of the patients with vaginal bleeding, UVA had been diagnosed by ultrasound, in three by ultrasound and MRI, in one by ultrasound and CT angiography (CTA), in one by ultrasound, MRI and CTA, in one by MRI only. In two emergent referrals from an outside hospital with ongoing bleeding after postpartal D&C no preinterventional cross-sectional imaging had been performed at our hospital (Table 2).

**Table 2:** Procedural characteristics and outcome

Pat	Age	Preinterventional imaging				Approach	Right uterine art	Left uterine art	Embolic agent	Secondary clinical success	Pregnancy	Birth
		TVUS	MRI	US	CT							
1	35	-	-	-	-	transarterial	selective	-	BCAL (1:3)	yes	yes	no
2	34	X	X	-	-	transarterial	-	selective	BCAL (1:2)	yes	yes	yes
3	36	-	-	-	-	transarterial	-	selective	BCAL(1:5)	yes	yes	yes
4	40	X	-	-	-	transarterial	selective	-	BCAL (1:4)	yes	yes	yes
5	32	X	X	-	X	transarterial	unselective	unselective	Gelatin sponge slurry	yes	yes	yes
6	38	X	-	-	-	direct perc. puncture	-	-	BCAL (1:4)	yes	yes	no
7	36	X	X	-	-	transarterial	selective	-	E. 500–700 μm	yes	yes	yes
8	20	X	-	-	X	transarterial	selective	-	E. 500–700 μm	yes	?	?
9	36	X	X	-	-	transarterial	selective	unselective	R: BCAL (1:3) L: E. 300-500 μm	yes	yes	yes
10	31	X	X	-	-	transarterial	-	selective	BCAL (1:3)	yes	no	no
11	34	-	-	X	-	transarterial	unselective	unselective	Gelatin sponge slurry	yes	no	no
12	35	-	X	X	-	transarterial	selective	selective	BCAL (1:1)	yes	no	no
13	32	-	X	-	-	transarterial	selective	selective	R: BCAL(1:3) L: Gelatin sponge slurry	yes	no	no

TVUS = Transvaginal ultrasound; BCAL = (2-butyl-cyanoacrylate:lipiodol); E. = trisacryl gelatin particles (Embospheres®)

Of the 13 patients included, at presentation four had had a vaginal delivery followed by a D&C of retained products of conception (30%), one a vaginal delivery (8%) and one a vacuum-assisted delivery (8%). Three women had had a spontaneous abortion (23%), two a medical (15%), one a surgical abortion (8%) and one a cesarean section (8%) (Table 1). Overall, 7 of the 13 patients had given birth to healthy children. Fertilization had been performed by intracytoplasmic sperm injection (ICSI) in 3 of the 13 patients.

Indication and intervention planning were discussed by an interdisciplinary team of interventional radiologists and obstetricians or gynecologists. The delay between the obstetric/gynecological event and the radiological intervention ranged from 19 to 193 days (median = 49 days).

A uterine artery branch pseudoaneurysm with venous shunting was detected by catheter angiography in 5 out of the 13 patients, whereas arteriolo-venous type of vascular lesions confined to the myometrium were found in 8 patients.

Embolization of the UVAs was performed in 12 patients via a retrograde approach using a 5-F sheath via the right common femoral artery. Both uterine arteries were catheterized for diagnostic angiography to depict the dominant supply of the UVA. No utero-ovarian anastomosis was detected in these angiograms.

Details regarding the embolization technique are listed in Table 2. If selective embolization was performed, a microcatheter was advanced as close as possible to the artery leading to the lesion. In non-selective embolization the embolic agent was delivered in the ascending trunk of the uterine artery.

Unilateral selective transcatheter embolization with 2-butyl-cyanoacrylate -lipiodol mixture (BCAL) (Histoacryl®, B. Braun Medical AG and Lipiodol® Ultra-Fluid, Guerbet) was performed in 5 of 12 patients. In two patients, unilateral selective embolization was performed with embolic trisacryl gelatin particles (500–700 μm Embosphere® Microspheres, Merit Medical systems Inc.). Two patients underwent selective BCAL embolization of one uterine artery branch, while for the contralateral uterine artery, non-selective embolization with trisacryl gelatin particles or gelatin sponge slurry (Spongostan ™, Ethicon, Johnson & Johnson) was performed. Bilateral non-selective transarterial embolization was performed with gelatine sponge slurry in two patients. One patient underwent bilateral selective embolization with BCAL. Finally, in one patient embolization of a uterine artery branch pseudoaneurysm was performed by percutaneous, ultrasound-guided, direct injection of BCAL mixture. That technique was chosen as at percutaneous sonography the pseudoaneurysm could well be depicted, was large, and had a neck. Further it looked easily percutaneously accessible.

The primary endpoints were the technical and clinical success rate as well as the post-interventional pregnancy and birth rate. Technical success was defined as no evidence of uterine UVA at final angiography. Clinical success was defined as cessation of heavy uterine blood losses for those patients with vaginal bleedings (*n* = 11) and eventual resumption of normal menstruations for the whole population treated.

## Results

The technical success rate was 100%. However, the primary clinical success rate was just 77% (10 out of 13 patients). In three patients re-embolization had to be performed due to persistent vaginal bleeding. In one patient who was initially treated with BCAL contralateral re-embolization with gelatin sponge had to be performed two hours after the first intervention. In one of the two patients, whose uterus was non-selectively embolized bilaterally with gelatine sponge slurry, re-embolization with trisacryl gelatin particles with a size of 500–700 μm of the left uterine artery and BCAL on the right side had to performed 31 days after the initial intervention. In the last patient of these three patients, who had undergone percutaneous direct embolization, a catheter angiography was obtained 9 days later because of persistent vaginal spotting. Although there was no evidence of any vascular uterine anomaly preemptive bilateral uterine artery gelatine sponge slurry embolization was performed. Approximately 2 years later, this patient had a spontaneous abortion with consecutive ongoing minor vaginal bleeding. The presumed source of hemorrhage, a uterine artery branch pseudoaneurysm, was re-embolized with gelatine sponge slurry via both uterine arteries without any clinical success. Eventually a further late re-embolization with BCAL was successful.

Overall procedures, no complications could be identified.

Long-term follow-up data were available for 12 out of the 13 treated patients as one woman could not be retrieved (median FU = 857 days (range 20–3651 days)).

Overall, the pregnancy rate was 8 out of 12 patients (66%) with a live birth rate of 6 out of 8 patients (75%). Of the 2 women who experienced a pregnancy without giving birth, one had a D&C for suspected embryonic malformation and the other had a spontaneous abortion. Amongst the 8 women who became pregnant after the uterine embolization, 6 had been treated with BCAL (selective transarterial, *n* = 5; direct puncture, *n*=1). The remaining two women with pregnancies after the uterine embolization had been treated by bilateral uterine artery gelatine sponge slurry embolization (*n* = 1) and unilateral trisacryl gelatin particles (*n* = 1).

**Fig. 1:**
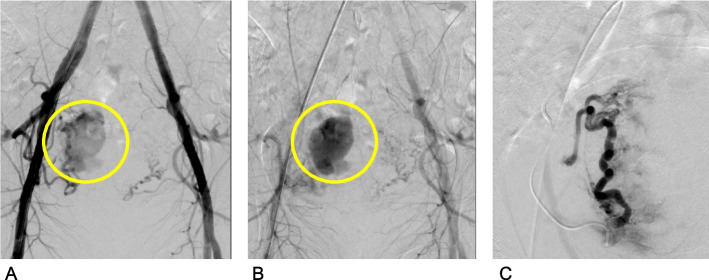
35-year-old patient. One month after a normal vaginal delivery a curettage was performed for recurrent bleedings assuming retained products of conception (RPOC). This procedure generated abundant vaginal blood losses. **A** and **B** Emergent pelvic angiography shows a pseudoaneurysm originating from the right uterine artery. **C** After selective embolization on the right side with BCAL (2-butyl-cyanoacrylate:lipiodol), there is no longer any perfusion of the pseudoaneurysm. Two years later an abortion curettage was performed

Of the two patients who became pregnant but did not give birth, both had been treated with BCAL, one unilaterally selectively (Fig. 1) and the other by percutaneous direct embolization.

## Discussion

This study shows that transarterial or direct embolization of acquired uterine vascular anomalies is a safe and effective treatment of vaginal blood losses related to acquired vascular uterine anomalies. According to the long-term outcomes observed, preservation of uterine function and eventually, further pregnancies, were likely not compromised by the embolizations.

The technical success rate of 100% and the clinical success rate of 77% in our study are in line with the current literature [2, 10–12]. Of the three patients who experienced early vaginal rebleeding after UAE one had been treated bilaterally, non-selectively, with gelatine sponge slurry, one with unilateral, selective BCAL, and one with percutaneous direct BCAL embolization. The technical and clinical success rate of UAE using a variety of embolic agents is reported to be high and has replaced traditional treatments such as hysterectomies or uterine artery ligations [12, 13]. The choice of the embolic material may be based on anatomical considerations (unilateral vs. bilateral uterine arterial supply of the UVA, accessibility of the UVA) or the operator’s personal experience.

The longer the delay between a pregnancy and the treatment, the more non-selective the delivery of the embolic agent will be due to the postpartal uterine shrinkage and the concomitant crowding of the uterine branches. Indeed, this non-selective embolic agent delivery could be related to failure in one of three patients, whose first embolization attempt was not curative. Direct percutaneous embolization seems an appealing alternative as it is faster when the culprit UVA can be depicted by ultrasound [14]. Unfortunately, the only woman we treated this way failed over time. She underwent early additional transarterial embolization and had a late transarterial re-embolization after an additional spontaneous abortion.

In our series, all women had a history of an obstetric event. Three patients had been fertilized by intracytoplasmic sperm injection (ICSI). Vaginal birth was responsible for about half of the UVAs in our patients, which is significantly higher compared to the literature. On the other hand, surgical, drug-induced or missed abortions as their etiology was slightly lower with a rate of 42% [2].

Based on a nationwide interrogation of 14 French experts in gynecology and interventional radiology appropriateness criteria for the treatment of acquired UVAs have recently been proposed [15]. Gelatine sponge slurry was preferred to microspheres or liquid embolic agents for most women with pregnancy plans, due to its complete absorption over time and lower risk of endometrial and ovarian ischemic complications. However, for recurrent bleedings after gelatine sponge slurry embolization no consensus was reached upon the use of microspheres or liquid agents as these embolic agents may be more efficacious, particularly for patients with hemodynamic complications. The choice of embolic agent had no influence on the technical and clinical success rate, and it does not appear to have had any influence on the pregnancy and birth rate in our study population. Of the 12 patients who were followed up, 8 became pregnant (66%) and 6 of them (75%) gave birth. Overall, BCAL was used in 9 out of 13 patients in our study population, but unilaterally applied in the UVA-feeding artery in 8 out of the 9 patients. This observation corroborates that the choice of the embolic agent, including “permanent” embolic agents like BCAL, does not hamper future fertility [3, 16, 17]. A recent systematic meta-analysis focused on pregnancy rates and outcomes of embolization for congenital and acquired AVMs included 44 pregnancies in 189 patients (10 case series and 18 case reports) [18]. In that review article the pregnancy rate after unilateral BCAL uterine artery embolization (up to 60%) tended to be higher than after bilateral uterine embolization with gelatine sponge or polyvinyl alcohol. These authors conclude that, overall, the life birth rate does not differ from that of a general population.

This study has some limitations. The sample size was small due to the rarity of the disease; additionally, women with variety of acquired UVAs and different etiologies were included. The choice of embolization techniques was not uniform due to the retrospective, uncontrolled study design. For instance, the choice of the embolic agent depended on the operator’s preference, too. These aspects may render any robust conclusions to be drawn difficult.

## Conclusion

In light of the low procedural complication rates, its high clinical success associated with remarkable postembolization pregnancy and birthrates rates, transarterial, possibly unilateral selective uterine embolization should be recommended for acquired UVAs treatment.

## Data Availability

The datasets used and/or analyzed during the current study are available from the corresponding author on reasonable request.

## Abbreviations

UVA: Uterine vascular anomalies
AVM: Arteriovenous malformations
UAE: Uterine arterial embolization
TAE: Transarterial embolization
DSA: Digital subtraction angiography
TVUS: Transvaginal pelvic ultrasound
D&C: Dilation and curettage
RPOC: Retained products of conception
ICSI: Intracytoplasmic sperm injection
BCAL: Butyl-Cyanoacrylate-Lipiodol Mixture
